# Crosstalk between interferon and interleukin-1 antiviral signaling in cancer cells: implications for immune evasion and therapeutic resistance

**DOI:** 10.3389/fimmu.2023.1219870

**Published:** 2023-06-08

**Authors:** Morten Frier Gjerstorff

**Affiliations:** ^1^ Department of Cancer and Inflammation Research, Institute of Molecular Medicine, University of Southern Denmark, Odense, ;Denmark; ^2^ Department of Oncology, Odense University Hospital, Odense, Denmark; ^3^ Academy of Geriatric Cancer Research (AgeCare), Odense University Hospital, Odense, Denmark

**Keywords:** interleukin 1, interferon, tumor immunity, therapeutic resistance, myeloid-derived suppressor cells (MDSC)

## Introduction

Mammalian cells have evolved defense systems to detect and respond to viral infections by producing cytokines that activate and shape the antiviral immune response (1, 2). Recent studies have revealed that cancer cells can also activate antiviral signaling in response to therapy-induced DNA damage and demethylation (3–6). This activation initiates the production of type I interferon (IFN), which is crucial for a T-cell response against cancer cells (4–6) and supports anti-tumor immunity (7). However, it has become apparent that therapy-induced activation of antiviral signaling can also trigger interleukin-1 (IL-1)-driven antiviral responses, which may not be advantageous as IL-1 promotes the production of cytokines that directly enhance tumor growth or inhibit anti-tumor immunity through the recruitment of myeloid-derived suppressor cells (MDSCs) to the tumor microenvironment (TME) (8). Therefore, it is important to understand the interplay between IFN and IL-1 antiviral responses in cancer cells and how this may impact immune evasion and anti-cancer therapy.

## IFN and IL-1 antiviral response pathways

The human body is constantly exposed to a wide range of pathogens, including viruses that can cause severe illnesses. To combat these pathogens, the immune system has evolved a complex network of defense mechanisms, including the antiviral response pathways (1, 2). The pathways are initiated by specialized receptors called pattern recognition receptors, which recognize viral components such as viral RNA or DNA. These receptors include surface molecules such as Toll-like and lectin receptors, as well as cytoplasmic receptors such as retinoic acid-inducible gene (RIG-I) and melanoma differentiation-associated gene 5 (MDA-5). The receptors are expressed in various cells, including epithelial and immune cells.

Activation of pattern recognition receptors triggers signaling cascades that converge into activation of IRF3 and IRF7 transcription factors and/or NF-κB pathways. This ultimately leads to the production of type I and III IFNs, which are cytokines that play a crucial role in the antiviral response (9, 10). Type I and III IFN bind to specific receptors on the surface of infected and neighboring cells, triggering a signaling pathway that results in the upregulation of a variety of antiviral effector molecules. The molecules can directly inhibit viral replication or induce apoptosis, thereby limiting the spread of the virus. In addition, the antiviral response leads to production of proinflammatory cytokines that support innate and adaptive immunity, including IL-1, which plays a crucial role in regulating the immune response to viral infections. Like IFNs, IL-1 contributes to the overall antiviral signaling by promoting local and systemic inflammation and activating immune cells (11). The expression of IL-1 is also mediated by IRF and NF-κB signaling pathways. Despite the important roles of both the IFN and IL-1 response pathways in the antiviral defense, the relationship between them remains poorly understood.

## Diverging roles of IFN and IL-1 antiviral pathways in cancer

Intriguingly, cancer cells can also activate antiviral signaling in the absence of viral infection through two distinct mechanisms: (i) the release of fragmented dsDNA into the cytosol due to genomic instability or (ii) epigenetic activation and transcription of dsRNAs from endogenous retroviruses (ERVs) in the genome (3–6). These mechanisms occur spontaneously in cancer cells due to their genomic and epigenetic instability and are strongly triggered by standard anti-cancer therapies such as radiation therapy, chemotherapy, and hypomethylating agents (3–6, 12–15). Activation of antiviral signaling in cancer cells can induce the expression of type I IFNs (3–6, 12–15), which generally inhibit cancer cell growth and support the function of immune cells with anti-cancer effects, such as T cells and NK cells (7). This suggests a beneficial role of spontaneous or therapy-induced activation of antiviral signaling in cancer cells.

**Figure 1:**
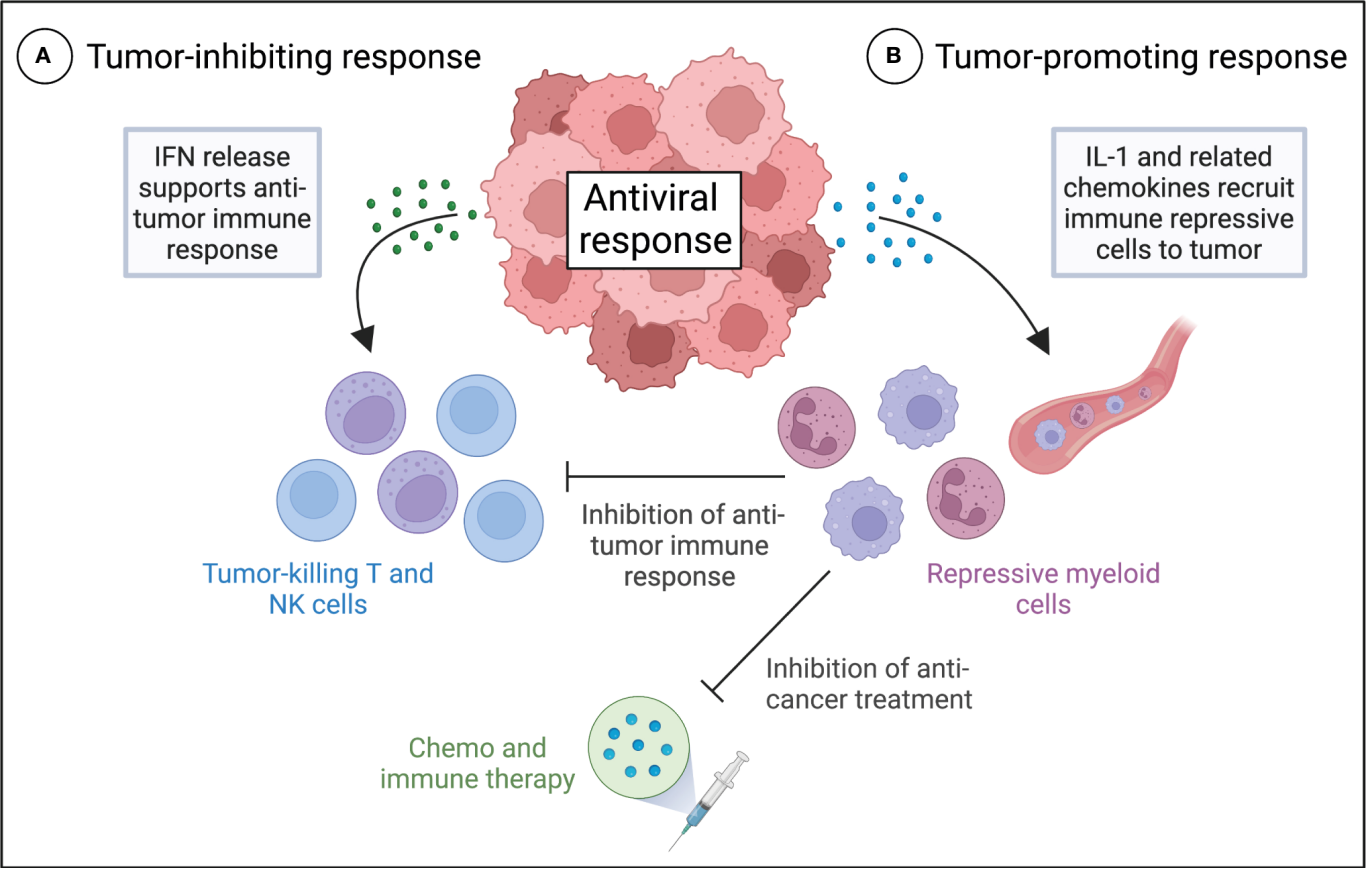
Different roles of IFN and IL-1 antiviral signaling in anti-tumor immunity and the therapeutic response of tumors. Proposed model for tumor inhibiting IFN-driven **(A)** and tumor promoting IL-1-driven **(B)** antiviral responses in cancer cells and their effects on the anti-tumor immune response and anti-cancer treatment. **(A)** Activation of IFN expression in tumors by the antiviral response results in autocrine and paracrine IFN signaling and production of a variety of antiviral effector molecules that support anti-tumor immunity. **(B)** Activation of IL-1 expression in tumors results in the production of multiple myeloid cell chemoattractant, which recruit MDSCs to the tumor microenvironment. MDSCs may restrict the activation, proliferation and functionality of T cells in the tumor microenvironment, leading to repression of anti-tumor immunity, and they may promote a poor response to various therapies, including chemotherapy, radiation, and immunotherapy.

However, recent research challenges the notion that IFNs are the primary drivers of antiviral responses in cancer cells. Evidence suggests that in a substantial subset of melanoma, breast and ovarian cancers, IL-1 is a potent moderator of the antiviral response to treatment with hypomethylating agents (8). In contrast to IFNs, IL-1 is considered to have unfavorable effects on anti-tumor immunity (11), largely due to its ability to enhance the recruitment of immune repressive cells to the TME (11, 16–24). In agreement, IL-1-driven antiviral response in cancer cells promote the expression of multiple myeloid cell chemoattractant, which recruit MDSCs to tumors (8). MDSCs are known to restrict the activation, proliferation and functionality of T cells in the TME, leading to repression of anti-tumor immunity (25–29). Additionally, tumor infiltration of MDSCs is associated with tumor progression and poor response to various therapies, including chemotherapy, radiation, and immunotherapy, across multiple tumor types (30). Therefore, many tumors may evade immune control and anti-cancer treatment by rewiring antiviral pathways to substitute a lethal IFN-driven inflammatory response with an IL-1-driven response (**Figure 1**).

## Interaction between IFN and IL-1 antiviral pathways

There is growing evidence to suggest that the interplay between IL-1 and IFNs is crucial in maintaining a delicate balance in the innate inflammatory response. Studies have shown that both IFN-α and IFN-β can downregulate the transcription of IL-1α and IL-1β, as well as inhibit the processing of the inflammasome that produces bioactive IL-1 (31–33). Additionally, numerous studies have shown that type I IFNs induce the expression of IL-1RA, which is an antagonist of the IL-1 receptor (34–36). These effects have also been observed in patients receiving type I IFN therapy (37). Thus, it appears that type I and III IFNs are capable of suppressing IL-1 activity at multiple levels.

The IFN-mediated regulation of IL-1 activity can have opposing effects during pathogen infections, depending on the situation. In some cases, the inhibition of IL-1 activity by IFNs can impair the host ability to mount a robust immune response against the pathogen. However, in situations where excessive IL-1 activity could lead to immunopathology, IFN-mediated suppression of IL-1 can be beneficial in limiting tissue damage and inflammation. Although much less studied, available data also suggest that IL-1 potently antagonizes type I IFN responses by directly regulating both transcription and translation of IFN-β (33, 38) as well as attenuating IFN-α/β-induced STAT phosphorylation (39).

Although limited, the available data suggest that there is crosstalk between IFNs and IL-1 in cancer as well. Specifically, it has been demonstrated that activation of antiviral signaling by DNA methyltransferase inhibitor-mediated de-repression of ERVs induce the expression of IFN and IL-1 genes in a complex pattern, with some cancer cell lines expressing either one alone or both together (8). This suggest that IFN and IL-1 antiviral responses are not mutually exclusive, despite the negative crosstalk between these signaling pathways. Additionally, it was found that DNA methyltransferase inhibitor-mediated induction of IL-1 was strongly suppressed by the presence of type I and III IFN, whereas IL-1 did not inhibit IFN expression. These findings suggests that there is interplay between IFN and IL-1 in cancer.

## Discussion

Antiviral signaling is increasingly recognized as a critical regulator of both tumor development and anti-tumor immunity. While much research has focused on the role of IFNs in this process (4, 5), recent evidence suggests that IL-1 may also play a significant role (8). Therefore, understanding the complex interplay between these two signaling pathways in tumors and their divergent roles in anti-tumor immunity is crucial.

For instance, it is well known that IFN signaling is often lost in cancer, but it is not known how this affects IL-1 activity and ultimately anti-tumor immunity. Therefore, studying the relationship between IFN and IL-1 in tumors will be essential to develop effective cancer therapies that activate antiviral signaling. It will also be important to comprehensively characterize the molecular differences that determine the relative activation IFN and IL-1 signaling in tumors. This understanding will provide profound biological insight into tumor biology and aid the identification of biomarkers for cancer patient stratification with respect to treatment with drugs that activate antiviral signaling, such as agents that induce DNA damage and demethylation.

Overall, elucidating the interplay between IFNs and IL-1 in cancer will have far-reaching implications for the development of novel cancer therapies and personalized medicine.

## Author contributions

MG wrote the manuscript.
